# Streaming Long‐Read Sequence Alignments for HLA Predictions Using HLAminer

**DOI:** 10.1002/cpz1.70124

**Published:** 2025-03-27

**Authors:** René L. Warren, Inanc Birol

**Affiliations:** ^1^ BC Cancer Genome Sciences Centre Vancouver BC Canada; ^2^ Department of Medical Genetics University of British Columbia Columbia BC Canada

**Keywords:** human leukocyte antigen (HLA), HLA typing, HLA inferences from sequencing data, long‐read sequencing for HLA prediction, next‐generation sequencing‐based HLA analysis

## Abstract

Long‐read sequencing platforms such as the Oxford Nanopore Technologies (ONT) and Pacific Biosciences (PacBio) platforms now offer sufficient read lengths, throughput, and accuracy at competitive costs to analyze polymorphic regions of the human genome, including the highly complex human leukocyte antigen (HLA) gene cluster—a cornerstone of human immunity. Here, we present a streamlined protocol for predicting HLA signatures from whole‐genome shotgun (WGS) long‐read sequencing data by directly streaming sequence alignments into HLAminer. This method is as simple as running minimap2, scales efficiently with the number of sequences, and works with any read aligner compatible with the SAM file format—eliminating the need to store bulky alignment files on disk. We provide a step‐by‐step guide for predicting HLA class I and class II alleles from third‐generation long‐read sequencing data and demonstrate the robustness of predictions even with older, less accurate WGS nanopore datasets and relatively low (10×) sequencing coverage. Code availability: HLAminer is available under the BC Cancer software license agreement (academic use) at https://github.com/bcgsc/HLAminer. © 2025 The Author(s). Current Protocols published by Wiley Periodicals LLC.

**Basic Protocol**: HLA prediction from streamed ONT or PacBio long‐read alignments

## Introduction

The landscape of commercially available nucleic acid sequencing technologies is changing rapidly (Athanasopoulou et al., 2022), and in recent years, long (N50 length ∼10‐20 kbp) and ultra‐long (>1 Mbp) DNA sequencing reads such as those offered by Pacific Biosciences of California, Inc. (PacBio; Menlo Park, CA) and Oxford Nanopore Technologies PLC (ONT; Oxford, UK) are becoming embedded in more genome sequencing projects (Mahmoud et al., 2024; Taylor et al., 2024; Wenger et al., 2019). This is in part because long reads are able to span large genomic repeats, enable allele phasing, and even provide methylation signals without the need for specialized or additional sample preparation (Akbari et al., 2022). Further, with the recently released Q20+ chemistry from ONT and CCS (HiFi) technology from PacBio, the base accuracy gap between long and short reads is shrinking, providing unprecedented opportunities for the analysis of genomes, including the characterization of challenging polymorphic gene loci such as that of the major histocompatibility complex (MHC).

The MHC, also known as the human leukocyte antigen (HLA) complex, is a multi‐gene locus comprising closely linked, highly variable genes that are critical to adaptive immunity. HLA genes are divided into two main classes: class I (HLA‐I: HLA‐A, HLA‐B, HLA‐C), encoding molecules presenting antigen‐derived epitopes to cytotoxic T cells (via CD8^+^ receptors), and class II (HLA‐II: HLA‐DQA1, HLA‐DQB1, HLA‐DRB1, HLA‐DPA1, HLA‐DPB1), encoding molecules that present epitopes to helper T cells (via CD4^+^ receptors). Together, these genes play essential roles in immune response and antigen recognition. Thousands of HLA alleles have been catalogued, with new ones being identified each year (Barker et al., 2023; Robinson et al., 2011). Each allele encodes a variant of the HLA cell surface receptor, characterized by its specificity for particular foreign‐derived antigen peptide epitopes. The presentation of these HLA epitopes to T cells is central to the human immune response, firmly establishing HLA as a critical determinant of disease (Dendrou et al., 2018). Furthermore, HLA typing is instrumental in understanding disease susceptibility and risk within population and patient cohorts (Warren & Birol, 2021; Warren et al., 2024). Clinically, determining a patient's HLA signature is vital for procedures such as organ transplantation, in which graft‐host compatibility is essential for success.

Over a decade ago, we pioneered the development of a bioinformatics approach for the automated prediction of HLA alleles from whole‐genome shotgun (WGS) sequencing, transcriptome sequencing (RNA‐seq) or whole‐exome sequencing (WES) short‐read datasets, alleviating the need for additional sample preparation(s), HLA sequencing kits, costs, and the specialized and manual labor typically required for the analysis of clinical HLA typing data (Warren et al., 2012). The approach, named HLAminer, originally relied on either targeted *de novo* sequence assembly or direct sequence alignments of Illumina‐sized short reads. Over time, other computational methods followed suit and new approaches emerged (Szolek et al., 2014; Lee & Kingsford, 2018), but HLAminer remained one of the few tools able to predict both class I and II HLA alleles, and do so from various sequencing data types (WGS, RNA‐seq, WES). Further, back in 2018 we adapted HLAminer (v1.4) to predict HLA alleles from any long sequence reads, by streaming sequence alignment outputs directly into it. Despite the rapid development of DNA sequencing technologies over the past two decades, only a few bioinformatics methods exist for HLA prediction from long sequencing reads, and reliable tools able to predict both HLA class I and class II alleles are still few (Liu, 2021; Wang et al., 2025).

Here, we present a straightforward, easy‐to‐execute protocol to predict HLA alleles from DNA sequencing datasets using up‐to‐date and continually expanding HLA sequence data from public repositories. This protocol enables the preparation of reference genome sequences for long‐read alignments and, using a single command at the Unix terminal, prediction of likely HLA class I and II alleles from whole‐genome long‐read sequencing data. To demonstrate and benchmark the HLAminer protocol presented herein, we utilized one PacBio HiFi and four ONT PromethION whole‐genome shotgun datasets derived from three individuals with known, clinically confirmed HLA types (Chin et al., 2020; Dilthey et al., 2016; Erlich et al., 2011; Lee & Kingsford, 2018). We anticipate that this single‐step streaming HLA prediction protocol will prove valuable to researchers and clinicians, particularly as nanopore sequencing facilitates real‐time DNA sequence data generation, and long‐read sequencing technologies increasingly permeate clinical applications.

## Strategic Planning

### Necessary Hardware

The HLAminer protocol requires a 64‐bit Linux or Mac operating system with sufficient random‐access memory (RAM) to process large volumes of DNA sequencing data (e.g., up to 35 GB RAM in our tests; see below). RAM requirements will depend on the properties of the data, including the sequencing read coverage, and the alignment parameters used. Both runtime and peak memory usage are determined by the sequence aligner used.

### Software Installation

This protocol uses HLAminer (v1.4, https://github.com/bcgsc/hlaminer; Warren et al., 2012), a command‐line script that requires the PERL interpreter—typically pre‐installed on most Linux and Mac systems—and the minimap2 read‐aligner (2.28‐r1209, https://github.com/lh3/minimap2; Li, 2018). We recommend manually cloning and compiling the HLAminer source code from GitHub and installing minimap2 using the conda package manager.

This protocol was tested with minimap2 (Li, 2018), as it is best in class for ONT and PacBio long‐read alignments at the time of this writing, but HLAminer will support any aligners capable of outputting a SAM (Sequence Alignment/Map)‐compatible output (i.e., .sam file) with MD tags (i.e., string encoding mismatched and deleted reference bases).

### Installing HLAminer

Installation by cloning the GitHub repository is recommended, as HLAminer is not a compiled code base and does not have any code or library dependencies. Cloning the repository is simply achieved using this command on the terminal:


git clone https://github.com/bcgsc/HLAminer/




*NOTE*: To run HLAminer from any directory, its executables must be accessible via the PATH environment variable. To achieve this, add the following line to your shell configuration file (e.g., .bashrc or .bash_profile), replacing /path/to/hlaminer/installation below with your corresponding installation directory:


export PATH=/path/to/hlaminer/installation/HLAminer/HLAminer‐1.4/bin:$PATH


Alternatively, for users running commands from the installation directory, the following approach ensures reproducibility and simplifies protocol execution:


export PATH=$(pwd)/HLAminer/HLAminer‐1.4/bin:$PATH
export HLAMINER_DBPATH=$(pwd)/HLAminer/HLAminer‐1.4/database



More detailed instructions for installing the packages can be found in their respective GitHub repositories.

### Installing minimap2

Installation via the Conda package manager (Miniconda) is recommended. Conda allows easy installation of tools and their dependencies into stand‐alone environments. Miniconda is a minimal, lightweight installation for Conda and can be obtained freely from https://docs.conda.io/en/latest/miniconda.html. Once Miniconda is installed, create a new environment for the protocol with the following commands:


conda create ‐n hlaminer python=3.8
conda activate hlaminer



minimap2 can then be installed from the bioconda channel:


conda install bioconda::minimap2



This command will install minimap2 into the hlaminer environment. This environment must be activated prior to running the streaming protocol with the command:


conda activate hlaminer



### Downloading HLA Sequence Data and Preparing the Reference Genome

HLAminer will work as‐is using the reference data files provided with the v1.4 distribution. However, new HLA alleles are continuously being discovered and reported and immunogenomics is an active field of research; the publicly available HLA sequence repositories that HLAminer relies on for its predictions must be updated for best results. To update the reference genome used for alignment, follow these steps at the terminal:

#### Option 1: Running the master download and formatting script




cd $HLAMINER_DBPATH
./updateAll.sh




#### Option 2: Executing the commands individually

1. Download genomic HLA sequences:

wget ftp://ftp.ebi.ac.uk/pub/databases/imgt/mhc/hla/fasta/*_gen.fasta

2. Concatenate HLA genomic sequences:

cat A_gen.fasta B_gen.fasta C_gen.fasta F_gen.fasta G_gen.fasta H_gen.fasta DP*_gen.fasta DQ*_gen.fasta DR*_gen.fasta | perl ‐ne ‘chomp;if(/\>\S+\s+(\S+)/){print “>$1\n”;}else{print “$_\n”;}’ > HLA‐I_II_GEN.fasta

3. Prepare a reference genome for long‐read sequence alignment:

cat GCA_000001405.15_GRCh38_genomic.chr‐only‐noChr6.fa HLA‐I_II_GEN.fasta | pigz ‐ > GCA_000001405.15_GRCh38_genomic.chr‐only‐noChr6‐HLA‐I_II_GEN.fa.gz

4. Update the p‐designation file:

rm ‐rf hla_nom_p.txt
wget http://hla.alleles.org/wmda/hla_nom_p.txt

#### Option 3: Downloading a static version of the reference genome with updated HLA genomic loci (as of January 2025)




wget https://www.bcgsc.ca/downloads/btl/hlaminer/GCA_000001405.15_GRCh38_genomic.chr‐only‐noChr6‐HLA‐I_II_GEN.fa.gz



#### Option 4: Downloading a static version of all reference files from Zenodo (dated January 27, 2025)

The static (January 2025) reference files we used in our protocol (below), and including hla_nom_p.txt, GCA_000001405.15_GRCh38_genomic.chr‐only‐noChr6‐HLA‐I_II_GEN.fa.gz, and HLA‐I_II_GEN.fasta, can be directly downloaded from Zenodo (https://doi.org/10.5281/zenodo.14751276).

When running any of the above commands, and when pigz/wget are not installed with your operating system, you may consider using gzip/curl instead, respectively.

*IMPORTANT*: To be successful, HLA predictions from direct read alignments must include all known HLA alleles and unrelated genomic regions in one reference (i.e., GCA_000001405.15_GRCh38_genomic.chr‐onlynoChr6‐HLA‐I_II_GEN.fa.gz) to help prevent spurious off‐target long‐read alignments to HLA alleles. For each option listed above, verify that the file GCA_000001405.15_GRCh38_genomic.chr‐only‐noChr6‐HLA‐I_II_GEN.fa.gz has been successfully written to /path/to/hlaminer/installation/HLAminer/HLAminer‐1.4/database and is not empty or corrupted.

## HLA PREDICTION FROM STREAMED ONT or PacBio LONG‐READ ALIGNMENTS

This Basic Protocol outlines how to run HLAminer on ONT or PacBio long‐read sequencing data in a single terminal command by streaming the output of minimap2 directly into the HLAminer.pl script. This is achieved using the commonly‐used Unix pipe operator (|), which connects the standard output (STDOUT) of the first process (minimap2) to the standard input (STDIN) of the second process (HLAminer in ‐a stream mode). We demonstrate the steps using several datasets, including ONT and PacBio long reads with varying fold coverage, as well as different ONT pore chemistries and base‐caller combinations (Table 1).

**Table 1:** Datasets Used for Protocol Demonstration

Dataset	Cell line or individual	Accession code or URL	Long‐read sequencing technology	Sequencing instrument	Info	Genome fold coverage (×)
A	NA19240	ERR2585115	ONT	PromethION	Guppy 1.4.0	10
B	NA12878	SRR10965087	ONT	PromethION	N/A	39
C	NA24385	SRX5327410	PacBio	Sequel	CCS	30
D	NA24385	https://labs.epi2me.io/gm24385_2021.05/	ONT	PromethION	Guppy 5.0.6, R9.4.1	67
E	NA24385	https://labs.epi2me.io/askenazi‐kit14‐2022‐12/	ONT	PromethION	Dorado, Kit14	72

*NOTE*: The software packages listed below and their dependencies must be installed and be referenced to in your PATH environment variable before you begin the protocol (see Strategic Planning).

*NOTE*: We illustrate the HLAminer streaming functionality on three cell lines (NA19240, NA12878, and NA24385/HG002) from human individuals with clinically typed HLA class I and II, provided for reference (Chin et al., 2020; Dilthey et al., 2016; Erlich et al., 2011; Lee & Kingsford, 2018). Nanopore long‐read WGS datasets for NA19240 (dataset A; ENA ERR2585115) and NA12878 (dataset B; ENA SRR10965087) and PacBio CCS data for individual NA24385 (dataset C; ENA SRX5327410) are used as demonstrations for HLA prediction below. We also demonstrated the application of the protocol to NA24385 guppy v5.0.6 (dataset D) and dorado kit14‐2022‐12 (dataset E) base‐called ONT datasets, downloaded as per the instructions provided in https://labs.epi2me.io/gm24385_2021.05/ and https://labs.epi2me.io/askenazi‐kit14‐2022‐12/, respectively (Table 1). Data for all five datasets are included in the tables below, but we only provide protocol steps for datasets A, B, and C, as the steps for both D and E are identical to those for A (i.e., ONT data).

### Necessary Resources

#### Hardware

Server or machine running a 64‐bit Linux or Mac operating system with sufficient RAM (up to 35 GB); we benchmarked our protocol on a dedicated server‐class CentOS Linux release 7.6.1810 system with 144 core Intel Xeon Gold 6150 CPUs at 2.70 GHz with 2.1 TB RAM.

#### Software

HLAminer v1.4 (https://github.com/bcgsc/HLAminer), commit:c00effa
Minimap2 2.28‐r1209 (https://github.com/lh3/minimap2)

#### Files

Long sequencing reads can be provided in compressed (.gz) or uncompressed FASTQ format; the reference genome described in Strategic Planning (under “HLA sequence data download and reference genome preparation”), e.g., GCA_000001405.15_GRCh38_genomic.chr‐only‐noChr6‐HLA‐I_II_GEN.fa.gz, should be provided as is.

#### Sample data

Nanopore long‐read WGS datasets for NA19240 (dataset A) and NA12878 (dataset B) and PacBio CCS data for individual NA24385 (dataset C), downloaded from the European Nucleotide Archive (ENA; https://www.ebi.ac.uk/ena) under accession codes ERR2585115, SRR10965087, and SRX5327410.

### HLA prediction from NA19240 data: ONT, dataset A

1a Install the HLAminer repository and minimap2 sequence aligner as outlined in the Strategic Planning section (above), and add all binaries to your PATH environment variable as instructed.
2a Update the reference genome sequence as outlined in Strategic Planning.
3a Create a new directory for running HLAminer on a given long‐read sequence‐data FASTQ file. Enter the new directory; soft‐link the reference genome, HLA sequence loci, and p‐designation files; and download long‐read data (shown below for NA19240):


mkdir NA19240
cd NA19240
wget ftp://ftp.sra.ebi.ac.uk/vol1/fastq/ERR258/005/ERR2585115/ERR2585115.fastq.gz
4a Run HLAminer in streaming (‐a stream) mode:


minimap2 ‐t 48 ‐ax map‐ont –MD $HLAMINER_DBPATH/GCA_000001405.15_GRCh38_genomic.chr‐only‐noChr6‐HLA‐I_II_GEN.fa.gz ERR2585115.fastq.gz | HLAminer.pl ‐h $HLAMINER_DBPATH/HLA‐I_II_GEN.fasta ‐s 500 ‐q 1 ‐i 1 ‐p $HLAMINER_DBPATH/hla_nom_p.txt ‐a stream
  Run time will vary based on the depth of coverage of the long‐read data. For this 10× dataset (dataset A), executing the workflow took 40 min (Table 2).
  When the run is complete, HLAminer will output two files, HLAminer_HPRA.csv and HLAminer_HPRA.log, corresponding to the final prediction comma‐separated variable (.csv) and log (.log) files, respectively.

/usr/bin/time ‐v ‐o minimap‐hlaminer‐NA19240_GENDBont.time minimap2 ‐t 48 ‐ax map‐ont –MD $HLAMINER_DBPATH/GCA_000001405.15_GRCh38_genomic.chr‐only‐noChr6‐HLA‐I_II_GEN.fa.gz ERR2585115.fastq.gz | HLAminer.pl ‐h $HLAMINER_DBPATH/HLA‐I_II_GEN.fasta ‐s 500 ‐q 1 ‐i 1 ‐p $HLAMINER_DBPATH/hla_nom_p.txt ‐a stream
  Preceded by the Unix time command (above), the protocol will capture run time and peak memory used in minimap‐hlaminer‐NA19240_GENDBont.time.
  For ONT reads (the current protocol steps), we recommend the use of the minimap2 preset ‐ax map‐ont.

**Table 2:** Protocol Resource Usage (Intel Xeon Gold 6150 CPU at 2.70 GHz, Using 48 Threads)

Dataset	Dataset size (GB, compressed)	Genome fold coverage (×)	Wall clock (hr:min)	Peak memory (GB)
A	26	10	0:40	30.1
B	107	39	2:21	30.9
C	68	30	1:14	29.3
D	161	67	7:06	29.9
E	177	72	6:09	33.9

### HLA prediction from NA12878 data: ONT, dataset B

1b Install the HLAminer repository and minimap2 sequence aligner as outlined in the Strategic Planning section, and add all binaries to your PATH environment variable as instructed.
2b Update the reference genome sequence as outlined in Strategic Planning.
3b Create a new directory for running HLAminer on a given long‐read sequence‐data FASTQ file. Enter the new directory; soft‐link the reference genome, HLA sequence loci, and p‐designation files; and download long‐read data (shown below for NA12878):


mkdir NA12878
cd NA12878
wget ftp://ftp.sra.ebi.ac.uk/vol1/fastq/SRR109/087/SRR10965087/SRR10965087_1.fastq.gz
4b Run HLAminer in streaming (‐a stream) mode:


minimap2 ‐t 48 ‐ax map‐ont –MD $HLAMINER_DBPATH/GCA_000001405.15_GRCh38_genomic.chr‐only‐noChr6‐HLA‐I_II_GEN.fa.gz SRR10965087_1.fastq.gz | HLAminer.pl ‐h $HLAMINER_DBPATH/HLA‐I_II_GEN.fasta ‐s 500 ‐q 1 ‐i 1 ‐p $HLAMINER_DBPATH/hla_nom_p.txt ‐a stream
  Run time will vary based on the depth of coverage of the long‐read data. For this 39× dataset (B), executing the workflow took 2 hr 21 min (Table 2).
  When the run is complete, HLAminer will output two files, HLAminer_HPRA.csv and HLAminer_HPRA.log, corresponding to the final prediction comma‐separated variable (.csv) and log (.log) files, respectively.

/usr/bin/time ‐v ‐o minimap‐hlaminer‐NA12878_GENDBont.time minimap2 ‐t 48 ‐ax map‐ont –MD $HLAMINER_DBPATH/GCA_000001405.15_GRCh38_genomic.chr‐only‐noChr6‐HLA‐I_II_GEN.fa.gz SRR10965087_1.fastq.gz | HLAminer.pl ‐h $HLAMINER_DBPATH/HLA‐I_II_GEN.fasta ‐s 500 ‐q 1 ‐i 1 ‐p $HLAMINER_DBPATH/hla_nom_p.txt ‐a stream
  Preceded by the Unix time command (above), the protocol will capture run time and peak memory used in: minimap‐hlaminer‐NA12878_GENDBont.time
  For ONT reads (the current protocol steps), we recommend the use of the minimap2 preset ‐ax map‐ont.

### HLA prediction from NA24385 data: PacBio, dataset C

1c Install the HLAminer repository and minimap2 sequence aligner as outlined in the Strategic Planning section, and add all binaries to your PATH environment variable as instructed.
2c Update the reference genome sequence as outlined in Strategic Planning.
3c Create a new directory for running HLAminer on a given long‐read sequence data FASTQ file. Enter the new directory; soft‐link the reference genome, HLA sequence loci, and p‐designation files; and download long‐read data (shown below for NA19240):


mkdir NA24385
cd NA24385

  Download all 39‐run accession FASTQ files from SRA at:


https://www.ebi.ac.uk/ena/browser/view/SRX5327410

  Concatenate all 39 subread FASTQ files into a single file: e.g.,


cat SRR*_subreads.fastq.gz > HG002_PacBio_CCS_all.fq.gz

4c Run HLAminer in streaming (‐a stream) mode:


minimap2 ‐t 48 ‐ax map‐hifi --MD $HLAMINER_DBPATH/GCA_000001405.15_GRCh38_genomic.chr‐only‐noChr6‐HLA‐I_II_GEN.fa.gz HG002_PacBio_CCS_all.fq.gz | HLAminer.pl ‐h $HLAMINER_DBPATH/HLA‐I_II_GEN.fasta ‐s 500 ‐q 1 ‐i 1 ‐p $HLAMINER_DBPATH/hla_nom_p.txt ‐a stream

  Run time will vary based on the depth of coverage of the long‐read data. For this 30× dataset (C), executing the workflow took 1 hr 14 min (Table 2).
  When the run is complete, HLAminer will output two files, HLAminer_HPRA.csv and HLAminer_HPRA.log, corresponding to the final prediction comma‐separated variable (.csv) and log (.log) files, respectively.

/usr/bin/time ‐v ‐o minimap‐hlaminer‐NA24385_GENDBpacbio.time minimap2 ‐t 48 ‐ax map‐hifi --MD $HLAMINER_DBPATH/GCA_000001405.15_GRCh38_genomic.chr‐only‐noChr6‐HLA‐I_II_GEN.fa.gz HG002_PacBio_CCS_all.fq.gz | HLAminer.pl ‐h $HLAMINER_DBPATH/HLA‐I_II_GEN.fasta ‐s 500 ‐q 1 ‐i 1 ‐p $HLAMINER_DBPATH/hla_nom_p.txt ‐a stream

  Preceded by the Unix time command (above), the protocol will capture run time and peak memory used in: minimap‐hlaminer‐NA24385_GENDBpacbio.time
  For PacBio CCS hi‐fi reads (the current protocol steps), we recommend the use of the minimap2 preset ‐ax map‐hifi.

## Commentary

### Background Information

We present a protocol for predicting the HLA signatures of human samples, by streaming associated WGS long‐sequencing‐read alignments directly into HLAminer. HLAminer was initially developed as a bioinformatics utility to predict HLA alleles *in silico* from various next‐generation (short‐sequencing‐read) shotgun‐sequencing data types, including WGS, RNA‐seq, and WES (Warren et al., 2012). A new streaming feature, supported by the input parameter ‘‐a stream’ and discussed in the Critical Parameters section, has been implemented to function seamlessly with both short‐ and long‐read data. The HLAminer protocol outlined here features a single‐step executable that simplifies HLA prediction by directly streaming the sequence alignments of long reads from popular sequencing technologies. Considering the volume of sequencing data to process, the streaming executable consumes a reasonable amount of RAM (<35 GB) and alleviates the need to store bulky sequencing read alignments to disk, thereby making the protocol accessible to groups with limited compute resources. The method is as simple as running minimap2 itself, is agnostic to sequence aligners as long as they output alignments in the SAM format (with MD tags), and is robust to relatively high base error and low sequence coverage. As more and more genomics projects include a long‐sequencing‐read data component, we expect HLAminer to continue providing valuable, robust HLA predictions and insights to the scientific community.

### Critical Parameters and Troubleshooting

When predicting HLA types from direct read alignments with HLAminer, the MD tag (e.g., minimap2 --MD) must be present in the resulting SAM alignment file (or stream), as otherwise HLA prediction output files are likely to be empty (Table 3). A low (<10×) sequencing read coverage may also be insufficient for predictions to be made, and lowering the minimum score threshold in HLAminer (‐s) may help resolve the issue of empty predictions. When joining the execution of a read aligner with HLAminer, HLAminer's ‐a stream must be specified. We recommend that users carefully inspect the logs, output files, and error codes if an error occurs. See Table 3 for a list of additional potential problems, along with their causes and solutions.

**Table 3:** Sources and Solutions to Potential Errors When Executing the HLAminer Protocol

Problem	Possible cause(s)	Solution
The .csv prediction file is empty	The input read depth is too low	Access or generate a sequencing dataset with more depth (ideally ≥30‐fold coverage)
	The score threshold(s) is/are too high	Lower the minimum score threshold
	The MD tag is absent from the alignment output	Ensure that you run minimap2 with the following: minimap2 ‐ax map‐ont --MD
	The database file GCA_000001405.15_GRCh38_genomic.chr‐only‐noChr6‐HLA‐I_II_GEN.fa.gz is empty or corrupt	Ensure that the database file is not empty and has been written to /path/to/hlaminer/installation/HLAminer/HLAminer‐1.4/database
Results obtained are different than presented here	New HLA alleles have been discovered since this study, which may skew the results	Download the version‐controlled reference files outlined in Strategic Planning under “HLA sequence data download and reference genome preparation,” option 4
	Tool versions may differ from those described herein	Ensure that the tool versions used match those listed in the protocol
HLAminer will not execute and the following error appears in the terminal window: Can't open ‐h NA for reading -- fatal	The ‐a stream was likely omitted	Ensure that the joint minimap2 and HLAminer command is properly formatted and that ‐a stream is included.
HLAminer terminates abruptly with the following error: Command terminated by signal 13	The Unix pipe is broken	You may silence the error by appending the HLAminer command with 2>/dev/null. To silence only the broken‐pipe warning, you may append it with this command instead: 2> >(grep ‐v 'terminated by signal 13' >&2)

### Understanding Results

The final output of HLAminer is a set of HLA class I and class II allele predictions for each major gene. The output file will have the suffix .csv. HLAminer will also log HLA inference results (suffix .log) as it proceeds through the prediction stages, to inform the user of its process. These output files are described in further detail at the HLAminer GitHub repository (https://github.com/bcgsc/HLAminer) and in the article describing HLAminer (Warren et al., 2012). Briefly, the .log file tracks the process of HLA mining and contains the following information:

- HLAminer command and parameters utilized;
- Read alignment output, sequence identity to HLA, and best HLA hit for each;
- Initial HLA allele summary, score, and expect value;
- Final summary, listing all predictions by highest score (i.e., most likely HLA allele).

The .csv file contains HLAminer predictions. Predictions are listed by HLA gene and ranked by highest score, with predictions for #1 and #2 representing the most likely HLA alleles (4‐digit resolution) for each major gene at the group (2‐digit resolution) level, respectively.

e.g.,


---------------------------------------------
SUMMARY
MOST LIKELY HLA‐I ALLELES (Confidence (‐10 * log10(Eval)) >= 30, Score >= 500)
Allele,Score,Expect (Eval) value,Confidence (‐10 * log10(Eval))
---------------------------------------------
HLA‐A
   Prediction #1 ‐ A*26
     A*26:33,22158.02,1.78e‐12,117.5
   Prediction #2 ‐ A*33
     A*33:24,12384.00,2.23e‐05,46.5
HLA‐B
   Prediction #1 ‐ B*15
     B*15:63,17000.00,7.64e‐08,71.2
   Prediction #2 ‐ B*55
     B*55:29,11362.00,1.77e‐05,47.5
HLA‐C
   Prediction #1 ‐ C*07
     C*07:02P,27076.00,3.08e‐11,105.1
   Prediction #2 ‐ C*04
     C*04:61,10630.00,1.18e‐08,79.3




For example, from these predictions, the individual is expected to be heterozygous with HLA‐I major gene A, groups A26 and A33, and alleles A*26:33 and A*33:24. The score, expect value and confidence are different representations of the same metric. The confidence represents the expect value (Eval) as a score (log_10_‐based, analogous to the phred score used to assess nucleotide base accuracy). Predictions are ambiguous when there are multiple predicted allele groups and/or protein‐coding alleles with the exact same score.

To evaluate HLA predictions with experimental long sequencing reads, we used HLAminer to identify the main HLA‐I and HLA‐II genes at the group and allele levels from four ONT and one PacBio HiFi human WGS datasets corresponding to three human individuals (NA12878, NA19240, and NA24385), directly from streamed minimap2 sequence alignments. These predictions closely matched clinically derived HLA types, demonstrating the tool's robustness even at lower sequencing coverage and with earlier base‐called datasets. Group‐level predictions matched all but one (94% accuracy) and three (88% accuracy) clinically derived types for HLA‐I and ‐II, respectively (Tables 4 and 5). Allele‐level predictions were largely consistent between HLAminer and clinically determined types for both HLA‐I and ‐II, and considering the low sequencing coverage (e.g., 10×) and low base accuracy of earlier base‐called dataset versions (guppy v1.4.0), they performed robustly well. High‐coverage (39×, 67×, and 72× for datasets B, D, and E, respectively) promethION datasets yield HLA class I and II predictions that are close to their clinically typed counterparts and were corroborated across sequencing runs and platforms (e.g., HLA‐I B*35:01 predicted from both ONTs and PacBio versus clinically typed as B*35:08, and HLA‐II DRB1*04:03P predicted from both ONTs and PacBio versus clinically typed as HLA‐II DRB1*04:02P, for NA24385). The run time for predictions varied between datasets and roughly scaled with the number of sequencing reads to align, with minimap2 being the primary pipeline bottleneck for both run time and RAM (Table 2).

**Table 4:** HLAminer HLA Class I Predictions from Human Whole‐Genome Shotgun Long Sequencing Reads Datasets Compared to Clinically Determined HLA Types

Dataset	HLA‐A	HLA‐B	HLA‐C
**NA19240, clinical**	**30:01/68:02**	**35:01/57:03**	**04:01/18:01**
NA12878, dataset A	31:16/68:02P	35:01P/57:03P	04:01P/18:01P
**NA12878, clinical**	**01:01/11:01**	**08:01/56:01**	**01:02/07:01**
NA12878, dataset B	01:140P/11:01P	08:01P/56:01P	01:02P/07:01P
**NA24385, clinical**	**01:01/26:01**	**35:08/38:01**	**04:01/12:03**
NA24385, dataset C	01:01P/26:01P	35:01P/38:01P	04:01P/12:03P
NA24385, dataset D	01:01P/26:214	35:01P/38:01P	04:01P/12:03P
NA24385, dataset E	01:01P/26:01P	35:01P/38:01P	04:01P/12:03P

The lowest‐numbered allele is indicated when two or more alleles are predicted for a group. HLA alleles are summarized using the p‐designation, where applicable. Clinically determined data are highlighted in boldface.

**Table 5:** HLAminer HLA Class II Predictions from Human Whole‐Genome Shotgun Long Sequencing Reads Datasets Compared to Clinically Determined HLA Types

Dataset	DQA1	DQB1	DRB1	DPA1	DPB1
**NA19240, clinical**	**01:02/05:01**	**05:02/03:01**	**16:02/12:01**	**02:01/02:02**	**01:01/01:01**
NA19240, dataset A	01:02P/05:01P	05:02P/03:01P	16:02P/12:01P	02:01P/02:02P	879:01P/01:01P
**NA12878, clinical**	**01:01/05:01**	**02:01/05:01**	**01:01/03:01**	**01:03/02:01**	**04:01/14:01**
NA12878, dataset B	01:01P/05:01P	01:01P/05:01P	01:02P/03:01P	01:03P/02:01P	445:01P/14:01P
**NA24385, clinical**	**01:01/03:01**	**05:01/03:02**	**10:01/04:02**	**NA**	**NA**
NA24385, dataset C	01:01P/03:01P	05:01P/03:02P	10:01P/04:03P	01:03P	04:01P/666:01
NA24385, dataset D	01:01P/03:01P	05:01P/03:02P	10:01P/04:03P	01:03P/02:01P	04:01P/23:01P
NA24385, dataset E	01:01P/03:01P	05:01P/03:02P	10:01P/04:03P	01:03P/02:02P	04:01P/23:01P

The lowest‐numbered allele is indicated when two or more alleles are predicted for a group. HLA alleles are summarized using the p‐designation, where applicable. Clinically determined data are highlighted in boldface.

### Author Contributions

**René L. Warren**: Conceptualization; data curation; formal analysis; investigation; methodology; project administration; resources; software; validation; writing—original draft; writing—review and editing. **Inanc Birol**: Funding acquisition; writing—review and editing.

### Conflict of Interest

The authors declare no conflict of interest.

## Data Availability

The example data used in our Basic Protocol are freely available. Nanopore long‐read WGS datasets for human cell lines NA12878 and NA19240 and PacBio CCS data for NA24385 were downloaded from the ENA (https://www.ebi.ac.uk/ena) using accession codes SRR10965087, ERR2585115, and SRX5327410, respectively. The NA24385 (HG002) ONT guppy and Kit14 dorado datasets were downloaded as per the instructions provided at https://labs.epi2me.io/gm24385_2021.05/ and https://labs.epi2me.io/askenazi‐kit14‐2022‐12/, respectively (Table 1). The static reference databases used in this protocol (dated January 27, 2025) can be downloaded from Zenodo (https://doi.org/10.5281/zenodo.14751276).
